# Establishment of a Patient-Derived Orthotopic Xenograft (PDOX) Model of HER-2-Positive Cervical Cancer Expressing the Clinical Metastatic Pattern

**DOI:** 10.1371/journal.pone.0117417

**Published:** 2015-02-17

**Authors:** Yukihiko Hiroshima, Yong Zhang, Nan Zhang, Ali Maawy, Sumiyuki Mii, Mako Yamamoto, Fuminari Uehara, Shinji Miwa, Shuya Yano, Takashi Murakami, Masashi Momiyama, Takashi Chishima, Kuniya Tanaka, Yasushi Ichikawa, Michael Bouvet, Takuya Murata, Itaru Endo, Robert M. Hoffman

**Affiliations:** 1 AntiCancer, Inc., San Diego, CA, United States of America; 2 Department of Surgery, University of California San Diego, San Diego, CA, United States of America; 3 Yokohama City University Graduate School of Medicine, Yokohama, Japan; 4 Department of Obstetrics and Gynecology, Kawasaki University Medical School, Kawasaki, Japan; National Cancer Institute, UNITED STATES

## Abstract

Squamous cell carcinoma of the cervix, highly prevalent in the developing world, is often metastatic and treatment resistant with no standard treatment protocol. Our laboratory pioneered the patient-derived orthotopic xenograft (PDOX) nude mouse model with the technique of surgical orthotopic implantation (SOI). Unlike subcutaneous transplant patient-derived xenograft (PDX) models, PDOX models metastasize. Most importantly, the metastasis pattern correlates to the patient. In the present report, we describe the development of a PDOX model of HER-2-positive cervical cancer. Metastasis after SOI in nude mice included peritoneal dissemination, liver metastasis, lung metastasis as well as lymph node metastasis reflecting the metastatic pattern in the donor patient. Metastasis was detected in 4 of 6 nude mice with primary tumors. Primary tumors and metastases in the nude mice had histological structures similar to the original tumor and were stained by an anti-HER-2 antibody in the same pattern as the patient’s cancer. The metastatic pattern, histology and HER-2 tumor expression of the patient were thus preserved in the PDOX model. In contrast, subcutaneous transplantation of the patient’s cervical tumors resulted in primary growth but not metastasis.

## Introduction

Cervical cancer is worldwide the second most common cancer in women with the majority of squamous cell carcinoma (SCC) [1] resulting in 454,000 cases and 200,000 deaths per year in 2010. Frequent metastatic sites are the pelvic lymph nodes, para-aortic lymph nodes, lung, extra-pelvic nodes, liver, and bones [2]. Approximately 11,000 new cases and 3,870 deaths occur for cervical carcinoma in the U.S. [3]. Stage and nodal metastasis are related to overall survival [4]. Chemotherapy drugs used for cervical cancer include: paclitaxel, carboplatin, cisplatinum, bleomycin, mitomycin-C, vincristine and irinotecan [5]. Retinoids and interferon, in combination with cytotoxic chemotherapy, have been shown to be effective [6]. However, there is no standard treatment for metastatic cervical cancer. Therefore, a patient-like mouse model of cervical cancer could be very useful.

Our laboratory pioneered the patient-derived orthotopic xenograft (PDOX) nude mouse model with the technique of surgical orthotopic implantation (SOI) [7–21]. Unlike subcutaneous-transplant patient-derived xenograft (PDX) models, PDOX models metastasize. Most importantly, the metastasis pattern correlates to the patient.

Histologically intact human colon-cancer specimens derived surgically from patients were implanted by SOI to the colon or cecum of nude mice. Extensive growth on the colon in 13 of 20 cases of implanted patient colon tumors was observed with subsequent regional, lymph-node, and liver metastasis, as well as general abdominal carcinomatosis [7].

SOI of histologically intact pancreatic-cancer specimens to the nude-mouse pancreas, resulted in a metastatic pattern that resembles the clinical pattern including local tumor growth, extending to the stomach and duodenum, metastases to the liver and regional lymph nodes, and distant metastases to the adrenal gland, diaphragm, and mediastinal lymph nodes. A 100% take rate was demonstrated for 5 cases, of a total 17 mice transplanted, 15 supported tumor growth. Immunohistochemical analysis of the transplanted human pancreatic tumors showed a similar pattern of expression tumor-associated glycoprotein 72 and carcinoembryonic antigen in the transplanted tumors and the original surgical biopsy [8].

Histologically-intact patient specimens of ovarian cancer were developed by SOI under the capsule of the nude mouse ovary. The tumors grew locally with a subsequent patient-like metastatic pattern, including the parietal peritoneum, colon, omentum, and ascites [10].

Histologically-intact patient breast tumor tissue was transplanted to the mammary fat pad of nude mice by SOI where the tumor tissue grew extensively and metastasized to the lung [11].

A patient-like metastatic model of human lung cancer constructed was developed with SOI via thoracotomy in immunodeficient mice [9]. Tumors were transplanted into the left lung in all these experimental animals. The left lung was used for tumor implantation for 2 reasons: (1) the loss of lung function is smaller in the left lung than in right-lung during surgery. The left-lung-operated animals survive the procedure better. (2) The left lung in mice has one lobe, enabling tumors to readily develop after implantation [9]. When a poorly-differentiated large-cell squamous-cell patient tumor 2268 was implanted to the left lung by SOI directly from surgery, 5 out of 5 mice produced locally-grown tumors, in an average time of 61 days. Opposite-lung metastases occurred, as well as lymph-node metastases. The primary tumors and metastases in the mice maintained their large-cell-squamous-cell morphology. When subcutaneously implanted tumors grew only locally in 2 of 4 animals and no metastases were observed [9].

In a clinical correlative study of 20 cases of stomach cancer that grew in nude mice, 5 had clinical liver metastases and all 5 cases resulted in liver metastases in the nude mice. Of the 20 cases, 6 had clinical peritoneal involvement of their tumor, and of these, 5 resulted in peritoneal metastasis in the nude mice. There were statistically significant correlations for both liver metastases and peritoneal involvement between patients and mice [12].

In the present report, we describe the development of a PDOX model of HER-2-positive cervical cancer with a metastatic pattern similar to the patient donor.

## Materials and Methods

### Animals

Female athymic (*nu/nu*) NCR nude mice (AntiCancer, Inc., San Diego, CA), 4–6 weeks old, were used in this study. Mice were kept in a barrier facility under HEPA filtration. Mice were fed with autoclaved laboratory rodent diet. All mouse surgical procedures and imaging were performed with the animals anesthetized by intramuscular injection of a 0.02 ml solution of 50% ketamine, 38% xylazine, and 12% acepromazine maleate. All animal studies were conducted with an AntiCancer Institutional Animal Care and Use Committee (IACUC)-protocol specifically approved for this study and in accordance with the principals and procedures outlined in the National Institute of Health Guide for the Care and Use of Animals under Assurance Number A3873–1.

### Specimen collection

The patient provided written informed consent and the tumor specimen was procured under the approval of the Institutional Review Board of University of California San Diego.

### Orthotopic and subcutaneous implantation of patient derived xenograft model of cervical cancer

Tumor tissues were obtained from the HER-2-positive cervical cancer patient at surgery and cut into fragments (3-mm^3^) and transplanted subcutaneously in nude mice both subcutaneously and orthotopically.

For orthotopic transplantation, a small midline incision (6- to 10-mm) was made on the lower abdomen of the mouse through the skin and peritoneum. The uterus was exposed through this incision, and a single tumor fragment (3-mm^3^) was sutured to the cervix of the uterus using 8–0 nylon surgical sutures (Ethilon; Ethicon Inc., NJ, USA). On completion, the uterus was returned to the abdomen, and the incision was closed in one layer using 6–0 nylon surgical sutures (Ethilon) [7, 13].

### Tissue histology

Tumor tissue was removed with surrounding normal tissues at the time of resection. The tissues were fixed in 10% formalin and embedded in paraffin before sectioning and staining. Tissue sections (3 μm) were deparaffinized in xylene and rehydrated in an ethanol series. Hematoxylin and eosin (H&E) staining was performed according to standard protocols. For immunohistochemistry, sections (5 μm) were then treated for 30 min with hydrogen peroxide (0.3%) to block endogenous peroxidase activity. The sections were subsequently washed with PBS and unmasked in citrate antigen-unmasking solution (Mitsubishi Kagaku Iatron, Inc., Tokyo, Japan) in a water bath for 40 min at 98°C. After incubation with 10% normal goat serum, the sections were incubated with anti-HER-2/ErbB2 (1:100; Cell Signaling Technology, Inc., Danvers, MA, USA) at 4°C overnight. The binding of primary antibodies was detected using anti-rabbit secondary antibodies and avidin/biotin/horseradish peroxidase complex (DAKO Cytomation, Kyoto, Japan) for 30 min at room temperature. The labeled antigens were visualized with the DAB kit (DAKO Cytomation). Finally, the sections were counterstained with hematoxylin and examined using an BH-2 microscope (Olympus Corp., Tokyo, Japan) equipped with a INFINITY1 2.0 megapixel CMOS digital camera (Lumenera Corporation, Ottawa, Canada). All images were acquired using INFINITY ANALYZE software (Lumenera Corporation) without post-acquisition processing.

### Statistical analysis

PASWStatistics 18.0 (SPSS, Inc) was used for statistical analyses. Correlations were examined using the Fisher’s exact test. A p value < 0.05 was considered statistically significant for all comparisons.

## Results and Discussion

### The PDOX, but not PDX, model of cervical cancer mimics the patient metastatic pattern

**Fig 1:**
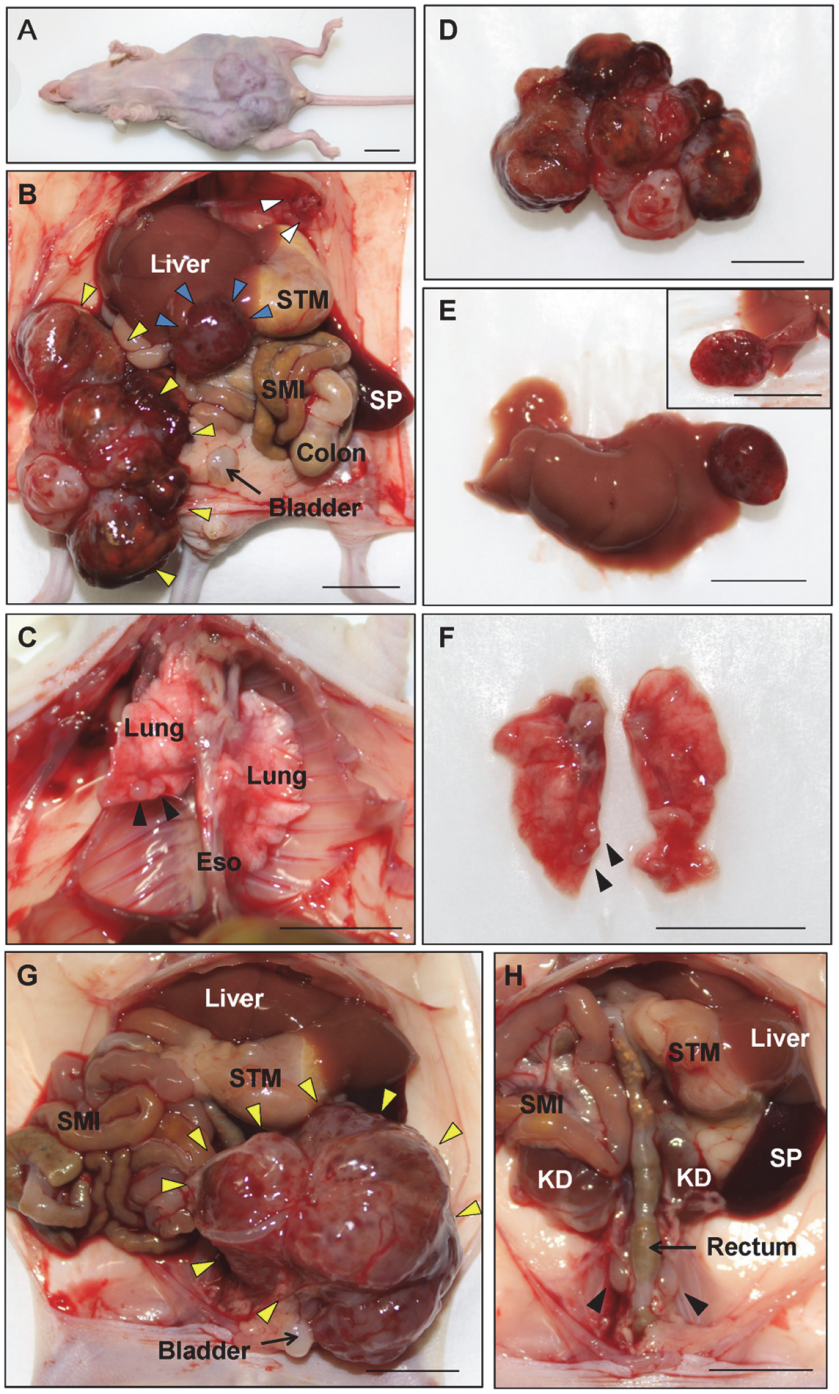
Metastasis in the cervical cancer PDOX model in nude mice. (A) Whole body imaging of the nude mouse orthotopically transplanted the patient’s cervical cancer specimen three monthly previously. The abdomen became distended with the tumor. (B) Images from laparotomy. The large primary tumor was fused with peritoneally-disseminated tumors (yellow arrows). Engorged nodule in the liver is a liver metastasis (blue arrows). Peritoneal dissemination was detected in the subphrenic space (white arrows). (C) Image thoracotomy. Two white nodules were detected in the lower lobe of the right lung (black arrows). (D) Excised specimens of primary tumor. The primary tumor was fused with peritoneally-disseminated tumors. (E) Excised liver metastasis specimens. Upper right inset shows the cut surface of tumor. (F) Excised lung metastasis specimens. Two white nodules were detected in the lower lobe of right lung (black arrows). (G and H) Images of a nude mouse developing para-aortic lymph node metastasis. The primary tumor engulfed the bladder (yellow arrows). After removal of the primary tumor, para-aortic lymph node metastases could be detected (black arrows). SP: spleen, STM: stomach, SMI: small intestine, Eso: esophagus, KD: kidney. Scale bars, 10 mm.

**Fig 2:**
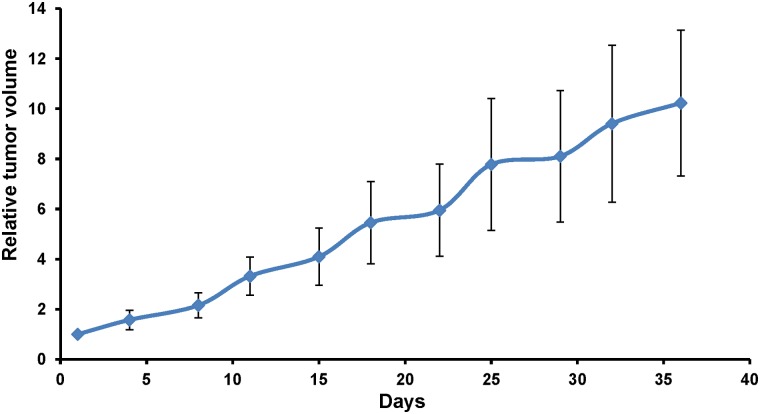
Growth rate of primary tumor. The tumor diameter was measured with calipers and volume calculated using the following formula: 4/3π●(d/2)^2^●D/2.

After subcutaneous transplantation in nude mice of the patient cervical tumor, tumor growth occurred in 7 of 10 mice but no metastasis occurred in any mouse (Table 1). Primary tumors grew in 6 out of 8 nude mice after orthotopic implantation and metastasis grew in 4 mice (Table 1). Metastasis in the nude mice included peritoneal dissemination, liver metastasis, lung metastasis, as well as para-aortic lymph node metastasis) (Fig. 1) (Table 2). The patient had metastasis in para-aortic lymph nodes, peritoneum, liver and messentary. Therefore, the PDOX model mimicked the patients tumors metastatic pattern and the subcutaneous PDX model had no metastasis. The growth rate of the primary tumor was rapid, doubling in 10–15 days during the 36-day growth period (Fig. 2). Examples of the sizes and shapes of the various metastasis on various organs were as follows: A roundish liver metastasis (8.5 mm × 8.3 mm) (Fig. 1E); two lung metastases (1.3 mm × 1.0 mm [oval]; 1.1 mm × 1.1 mm [round]) (Fig. 1F); and two para-aortic lymph node metastases (4.0 mm × 2.3 mm [oval]; 4.6 mm × 2.1 mm [oval]) (Fig. 1H). In future experiments, we will monitor the growth of metastasis from the cervical carcinoma by intravital as well as non-invasive imaging [15–17].

**Table 1:** Comparison of primary tumor and metastasis occurrence in subcutaneous (PDX) and orthotopic (PDOX) models.

Model	No. of mice implanted	Tumor-take rate (%)	Metastasis (%)
Nude mouse subcutaneous (PDX)	10	7 / 10 (70)	0 (0)
Nude mouse orthotopic (PDOX)	8	6 / 8 (75)	4 / 8 (50)

**Table 2:** Sites of metastasis in PDOX model and the patient.

Mouse	Sites of metastasis
Mouse 1	Peritoneum, Liver, Lung
Mouse 2	Peritoneum, Mesentery
Mouse 3	Peritoneum
Mouse 4	Para-aortic lymph nodes, Mesentery
Patient	Para-aortic lymph nodes, Peritoneum, Mesentery, Liver

### Histology of the original tumor is preserved in the mouse

**Fig 3:**
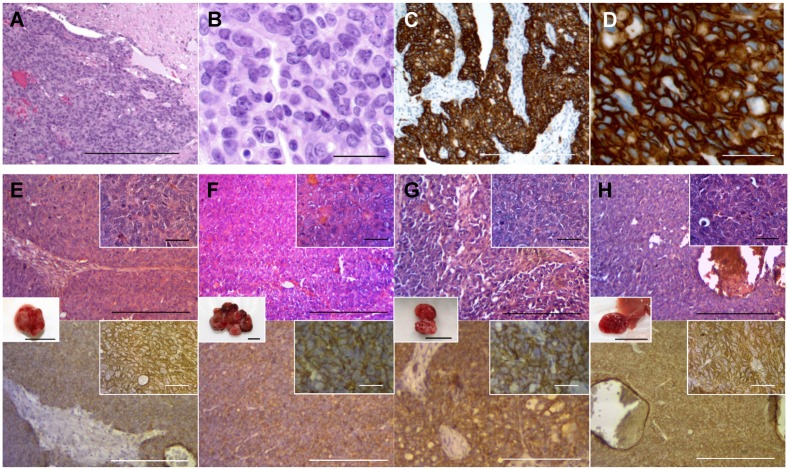
Histology of the patient and mouse-grown tumors and metastasis. (A) H&E-stained section of the original patient tumor. (B) High magnification image of (A). (C) Immunostained section of the original patient tumor using anti-HER-2 antibody. (D) High-magnification image of (C). (E-H) H&E-stained and immunostained sections of mouse-grown tumors. Upper panels are H&E-stained sections and lower panels are immunostained sections using an anti-HER-2 antibody. Right upper insets are high magnification images (scale bars: 25 μm) and the insets between upper and lower panels are images of the tumors (scale bars: 10 mm). All mouse-grown tumors, including the subcutaneous tumors (E), primary orthotopic tumor (F), peritoneal-disseminated metastasis (G) and liver metastasis (H) had histological structures similar to the original patient tumor and were stained by an anti-HER-2 antibody. Scale bars: 200 μm (A, C, E—H) and 25 μm (B and D).

Sheet-like growth without gland formation and stromal tissue with fibroblastic proliferation, which penetrated into the nests of carcinoma were observed in the H&E stained sections of the original tumor (Fig. 3A). Oval- to spindle-shaped cancer cells with high nuclear/cytoplasmic ratio were found in high magnification images (Fig. 3B). In the immunostained sections with anti-HER-2 antibody, the membrane and the cytoplasm of cancer cells were strongly stained but no staining was found in the stromal tissue (Fig. 3C and 3D). All mouse-grown cervical cancer patient tumor including the metastatic tumors had histological structures similar to the original tumor and were stained by anti-human HER-2 antibody (Fig. 3E-3H), suggesting that the model recapitulates the biological behaviors of the original tumor.

A PDOX model of HER-2-positive metastatic cervical cancer, which shows metastatic behavior throughout the mouse body including liver, lymph-nodes, lung, peritoneum, has not been previously reported. More important, the metastasis pattern of the PDOX tumor mimicked that of the patient. We further demonstrated that the mouse-grown tumors recapitulate the character of the original patient tumor by examining the expression status of HER-2. In the present study, we demonstrate that primary tumors and metastasis grown in the mouse were stained by anti-HER-2 antibody and recapitulated histological structures of the original tumor (Fig. 2). The incidence of HER-2 positivity in cervical cancer was reported from 1% to 21% [22], and overexpression of HER-2 has been associated with more advanced stages and a worse prognosis [23, 24].

The PDOX model of cervical cancer described in the present report could have multiple uses: For example, we have recently used a PDOX nude mouse model of pancreatic cancer and colon cancer to develop techniques for fluorescence-guided surgery [25–27]. A pancreatic cancer PDOX nude mouse model was used to evaluate the efficacy of the tumor-targeting *Salmonella typhimurium* (*S*. *typhimurium*) A1-R strain we are developing. *S*. *typhimurium* A1-R significantly reduced tumor growth compared to the untreated control [28].

*S*. *typhimurium* A1-R, in combination with anti-vascular endothelial growth factor (VEGF) therapy using bevacizumab (BEV), was active in the pancreatic cancer PDOX [29].

We also previously demonstrated zoledronic acid (ZA) is active on pancreatic cancer in a PDOX model on primary and metastatic growth [30].

Thus, the patient-mimicking PDOX model of cervical cancer described in the present report presents many opportunities for discovery of novel effective therapeutics for this currently treatment-resistant disease. After discovery of more effective therapeutics, the PDOX model of cervical cancer can be more useful for individualized patient therapy. The present report also emphasizes the critical advantages of the PDOX model over the subcutaneous PDX model [31–34].
